# Targeting GRP75 Improves HSP90 Inhibitor Efficacy by Enhancing p53-Mediated Apoptosis in Hepatocellular Carcinoma

**DOI:** 10.1371/journal.pone.0085766

**Published:** 2014-01-17

**Authors:** Weiwei Guo, Lichong Yan, Ling Yang, Xiaoyu Liu, Qiukai E, Peiye Gao, Xiaofei Ye, Wen Liu, Ji Zuo

**Affiliations:** Department of Cellular and Genetic Medicine, School of Basic Medical Sciences, Fudan University, Shanghai, China; Taipei Medical University, Taiwan

## Abstract

Heat shock protein 90 (HSP90) inhibitors are potential drugs for cancer therapy. The inhibition of HSP90 on cancer cell growth largely through degrading client proteins, like Akt and p53, therefore, triggering cancer cell apoptosis. Here, we show that the HSP90 inhibitor 17-AAG can induce the expression of GRP75, a member of heat shock protein 70 (HSP70) family, which, in turn, attenuates the anti-growth effect of HSP90 inhibition on cancer cells. Additionally, 17-AAG enhanced binding of GRP75 and p53, resulting in the retention of p53 in the cytoplasm. Blocking GRP75 with its inhibitor MKT-077 potentiated the anti-tumor effects of 17-AAG by disrupting the formation of GRP75-p53 complexes, thereby facilitating translocation of p53 into the nuclei and leading to the induction of apoptosis-related genes. Finally, dual inhibition of HSP90 and GRP75 was found to significantly inhibit tumor growth in a liver cancer xenograft model. In conclusion, the GRP75 inhibitor MKT-077 enhances 17-AAG-induced apoptosis in HCCs and increases p53-mediated inhibition of tumor growth *in vivo*. Dual targeting of GRP75 and HSP90 may be a useful strategy for the treatment of HCCs.

## Introduction

Hepatocellular carcinoma (HCC) is one of the most common malignant tumors and the third leading cause of cancer death worldwide [1]. Although surgical resection and liver transplantation can be curative in early-diagnosed cases or in patients with localized tumors, approximately 80% of patients with advanced liver cancer must largely rely on conventional chemotherapies [2], which have limited effectiveness due to the development of drug resistance and undesirable side effects. For this reason, new and effective therapeutic strategies for HCC are urgently needed.

Heat shock family proteins, particularly heat shock protein 90 (HSP90), constitute promising therapeutic targets for a broad range of cancers [3], [4]. Overexpression of HSP90 in cancer tissues, including HCC, has been shown to correlate with poor prognosis and poor treatment outcomes [5], [6]. Moreover, in cancer cells, increased concentrations of HSP90 play a significant role during the early stages of oncogenesis and in the maintenance of the malignant cell phenotype [3]. For these reasons, several small molecular weight inhibitors of HSP90 have been developed as potential antitumor agents for use against solid tumors, including various neuromas and hematologic malignancies [7]. To date, several HSP90 inhibitors have been identified and many of which are being currently tested in clinical trials (NCT01602627, NCT01259089; www.clinicaltrials.gov) [8]–[10]. 17-allylamino-17-demethoxy-geldanamycin (17-AAG), which was the first HSP90 inhibitor entered clinical trials, has been shown to induce apoptosis in cancer cells in a p53-dependent manner [11]. In addition, 17-AAG has been shown to stimulate dephosphorylation of Akt (protein kinase B), thereby reducing Akt stability and its ability to promote cell survival [12]. The activation of Akt depends on two phosphorylated sites: the threonine 308 and serine 473 [13].

GRP75 (glucose-regulated protein 75), a molecular chaperon belonging to the heat shock protein 70 family, also known as mortalin, peptide binding protein 74 (PBP74) and mitochondrial heat shock protein 70 (mthsp70), is ubiquitously enriched in mammalian cells and implicated in multiple biological functions, including stress responses [14], mitochondrial biogenesis [15], and differentiation [16]. Increased expression of GRP75 has been reported in several cancers, including leukemia [17], brain cancer [18], colorectal adenocarcinoma [19], and hepatocellular carcinoma [20]. Elevated expression of GRP75 in colorectal adenocarcinomas was correlated with malignant transformation and poor patient survival [21]. Furthermore, higher serum GRP75 level has shown to be associated with rapid disease progression and a risk factor in patients with colorectal cancer [21]. Overexpression of GRP75 in liver cancer was correlated with metastasis and early tumor recurrence [21]. In addition, It has been shown that overexpression of GRP75 was sufficient to increase the malignancy of breast carcinoma cells [22]. To a large part, upregulation of GRP75 increasing the malignancy of tumors results from its ability to bind cytoplasmic p53 [23]. Increased level of GRP75 in response to stresses such as reactive oxygen species and chemotherapy drugs treatment combines with and sequesters p53 in the cytoplasm, thereby impairing p53-dependent transcriptional activation function and promotion of apoptosis [24], [25]. These data identify GRP75 as a potential candidate target for cancer therapeutics. Selective knockdown of GRP75 expression by RNA interference induces cell growth arrest and enhances cell apoptosis [14], [26].

Also, it has been shown that the GRP75 inhibitor MKT-077, a cationic rhodacyanine dye analogue, exerted its growth inhibitory effect on cancer cells through disrupting GRP75-p53 interactions, promoting p53 nuclear translocation and restoring p53 transcriptional activity and apoptosis [24], [27].

In this study, we investigated whether GRP75 inhibition can enhance the anti-tumor effects of HSP90 inhibitor. Our results provide the first evidence for possibly enhanced HSP90-based antitumor therapy through co-inhibition of the p53 cofactor GRP75.

## Materials and Methods

### Ethics Statement

This study was approved by the Ethics Committee of Affiliated Hospital of Nantong University (Jiangsu), and each participant signed an informed consent document. Human HCC and non-tumor liver tissue samples were obtained from patients undergoing surgical treatment at Affiliated Hospital of Nantong University in accordance with the appropriate institutional review boards. All animal experiments and procedure were approved by the Committee for Animal Care and Use, Fudan University.

### Cell Culture and Reagents

Human liver cancer cell lines Bel-7402, HuH-7, and Hep3B (p53 null) were obtained from ATCC. Bel-7402, HuH-7 cells were cultured in Dulbecco’s Modified Eagle’s Medium (Gibco), and Hep3B cells were cultured in Minimum Essential Medium (Gibco), with 10% fetal bovine serum at 37°C with 5% CO_2_ in humidified atmosphere. The HSP90 inhibitor 17-N-allylamino-17-demethoxygeldanamycin (17-AAG; Biovision, Milpitas, CA, U.S.) was dissolved in DMSO (dimethyl sulfoxide) (1 mM). MKT-077 (Sigma, St. Louis, MO, USA) was dissolved in physiological saline (1 mg/mL).

### Immunohistochemisty Staining

Tissue assays were assembled and IHC-stained for GRP75 and HSP90 expression according to standard protocols. Tissue slides were incubated with rabbit anti-human GRP75 (1∶50; Cell Signaling Technology, Boston, MA, U.S. Lot No.3593S) and rabbit anti-human HSP90 mAb (1∶100; Epitomics, Billerica, MA, U.S. Lot No.3363-1) at 4°C overnight followed by incubation with anti-rabbit HRP-conjugated antibodies and detection using a 3,3′-diaminobenzidine (DAB) staining system. The slides were counterstained with hematoxylin. The stained slides were observed microscopically and images acquired using a microscope (Leica, CMS GmbH) and all pictures were taken using a Cool Snap CCD camera (Leica, DFC 300 FX) attached to the microscope.

### Cell Viability Assay

HCC cells were seeded at a density of 5000 cells per well in 96-well plates. After 24 hours, these cells were exposed to 17-AAG, MKT-077, or 17-AAG+MKT-077 for another 24 hours. For cell viability assays, 10 *µ*L CCK-8 solution (Cell Counting Kit-8, Dojindo Laboratories, Japan) was added to 100 *µ*L of medium per well. Absorbance was measured at 450 nm after the cells had been incubated for 2 hours.

### FACS Analysis

After the cells were treated, Annexin V/PI assays (BD Biosciences, San Diego, CA, U.S.) were performed to detect cell death. Briefly, the cells were trypsinized and diluted to 1×10^6^ cells/mL in 1×binding buffer (0.1 M Hepes/NaOH (pH 7.4), 1.4 M NaCl, 25 mM CaCl_2_). Then 6 *µ*L Annexin V and 6 *µ*L PI (propidium iodide) were added to 120 *µ*L cell suspension. After incubation for 15 min at room temperature in the dark, the cells suspension were combined with 480 *µ*L 1×binding buffer and analyzed using a FACSCalibur Flow Cytometer (Becton Dickinson, Franklin Lakes, NJ, U.S.).

### Western Blotting

The whole-cell lysates, nuclear/cytoplasm extracts, and subcutaneous tumor lysates were prepared as described [28]. Then 50 *µ*g protein samples were resolved by 10% SDS-polyacrylamide gel electrophoresis and electrophoretically transferred to polyvinylidene fluoride membranes (Millipore Corporation, Billerica, MA, U.S.). Membranes were incubated with antibodies specific for cleaved PARP (Lot No.1074-1), Akt (Lot No. 1081-1), phospho-Akt^Ser473^ (Lot No. 2118-1), phospho-Akt^Thr308^ (Lot No.2214-1) (Epitomics, Billerica, MA, U.S.), GRP75 (Lot No.3593S), p53 (LKtag, Shanghai, China, Lot No. 3020), phospho-p53^Ser15^ (Lot No. 9284S), and phospho-p53^Ser37^ (Lot No.9289) (Cell Signaling Technology, Danvers, MA, U.S.), and GAPDH (Lot No. G8795), α-tublin (Lot No. 926576) (invitrogen, Grand, NY), and LaminB1 (Abcam, USA, Lot No. ab16048) served as loading controls. Bound antibodies were visualized with Enhanced Chemiluminescence Reagent (Pierce Biotechnology Inc., Rockford, IL, U.S,).

### Co-immunoprecipitation

Cell lysates containing 500 *µ*g protein were incubated with the anti-GRP75 antibodies for 1 hour at 4°C. Then 40 *µ*L protein A/G agarose beads (Bioworld) were added and the mixtures allowed incubating at 4°C with gentle rocking overnight. The supernatants were aspirated and discarded and the beads washed three times with PBS. After the final wash, proteins were denatured by boiling in 40 *µ*L 1× SDS-PAGE sample buffer. Immunoblotting was performed using anti-GRP75, anti-p53, anti-Akt, and anti-HSP90 antibodies, as indicated.

### Immunofluorescence Staining

Cells were seeded on glass-bottom 24-well plates, and fixed with 4% paraformaldehyde in PBS, prior to permeabilization of the cell membranes with 0.01% saponin for 30 min, staining of the nuclei with Hoechst 33258 (Sigma, St. Louis, MO, USA) and immunostaining for p53 (Epitomics, Billerica, MA, U.S. Lot No. 1047-1) as described previously described [29].

### Quantitative Real-time RT-PCR

Total RNA from Bel-7402 cells was isolated using Trizol and 10 *µ*g of total RNA was reverse-transcribed using oligo dT primers and reverse transcriptase (Thermo, Waltham, MA, U.S.). The primer sequences used quantitative real-time PCR were as follows: p21-F: GGAGACTCTCAGGGTCGAAAACG, R: CGGATTAGGGCTTCCTCTTGGAG; PUMA-F: ACGACCTCAACGCACAGTACGAG, R: GTAAGGGCAGGAGTCCCATGATG; and MDM2-F: GCAGGGGAGAGTGATACAGATTC, R: AATGTGATGGAAGGGGGGGATTC, GRP75-F: AGCTGGAATGGCCTTAGTCAT, R: CAGGAGTTGGTAGTACCCAAATC. The relative expression of all these target genes of p53 was normalized to GAPDH expression, which served as an internal efficiency control. Amplification and detection of the products were carried out in a Mini opticon real-time PCR system (Bio-Rad, CA, USA) under the following protocol: at 94°C for 30 s, 58°C for 40s, and 38 cycles.

### Xenograft Assays

Five-to-six-week-old athymic female nude mice (nu/nu) were purchased from the Institute of Zoology (Chinese Academy of Science). Bel-7402 cells were suspended in phosphate buffer solution (PBS) and injected subcutaneously into the right flanks of the mice at 5×10^6^ cells per flank. Tumor volumes were measured using the following formula: length×width^2^×0.5. Drug treatment was continued until the tumor volume reached a value between 200 and 300 mm^3^. Tumors were measured every-other day. Mice were randomly divided into four groups: control, MKT-077, 17-AAG, and MKT-077+17-AAG. Treatment with MTK-077 and 17-AAG was performed by intraperitoneal (IP) injection at doses of 3 mg/kg and 80 mg/kg, respectively. MKT-077 was injected every-other day, and 17-AAG was injected on days 1–4 and 9–12. The mice were sacrificed 14 days after injection, when the tumors in the control group reached 1000 mm^3^. Tumor tissues were isolated in RIPA buffer (Cell Signaling Technology, Danvers, MA, U.S. Lot No. 9806,) containing Protease Inhibitor Cocktail (Roche) and detected by immunoblotting.

### Statistics

The statistical significance of differences between groups was assessed using GraphPad Prism 5.0 software. The unpaired 2-tailed *t* test was used for parameters between groups, and the level of significance was set at a *P* value of <0.05. Data are shown as mean ± SEM unless otherwise noted.

## Results

### GRP75 and HSP90 Overexpression in HCCs

**Figure 1:**
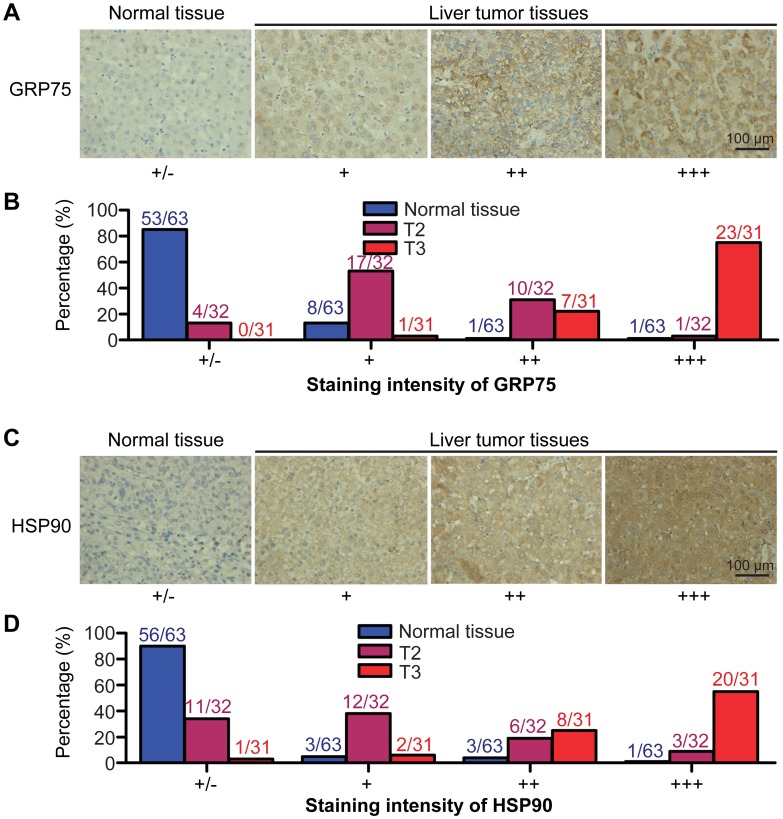
Overexpression of GRP75 and HSP90 in HCC tissues. Tumor tissue arrays containing 63 pairs of non-tumor and HCC tissues were stained with GRP75 and HSP90 specific antibodies using a DAB detection kit. (A, C) Representative images of immunohistochemically stained GRP75 or HSP90 proteins in paraffin-embedded non-tumor liver and liver tumor tissues. Normal and tumor tissues were classified into four groups based on staining intensities. (B, D) Tabulation of the percentage of normal, T2 and T3 cells within each group. 32 from pathologic stage T2 patients and 31 from T3 patients, tumor staging was determined according to the sixth edition of the TNM (tumor-node-metastasis, TNM) classification of International Union Against Cancer.

To determine the clinical significance of GRP75 and HSP90 in liver cancer, we evaluated the expression of GRP75 and HSP90 in HCC tissues and adjacent noncancerous tissues by immunohistochemically staining human HCC tissue arrays with anti-GRP75 and anti-HSP90 antibodies. These arrays comprised 63 primary liver tumor tissues [32 from pathologic stage T2 patients and 31 from T3 patients; classified based on the International Union Against Cancer’s Tumor-Node-Metastasis (TNM) Classification System (Sixth Edition)] and adjacent noncancerous liver tissues. As shown in Figure 1A and C, GRP75 and HSP90 were expressed weakly in normal tissues and overexpressed in HCC tissues. To determine the degree to which HCC tissues overexpressed GRP75 and HSP90, we divided the samples into four groups based on staining intensity from weakest (+/−) to strongest (+++; Figure 1B, D). As summarized in Figure 1B and D, the expression of GRP75 and HSP90 was very weak in the majority of non-tumor liver tissues, with 85% and 90% samples being placed in group 1. In contrast, GRP75 and HSP90 staining was very high in HCC tissues, and most of these were placed in groups 3 or 4. These data confirmed that GRP75 and HSP90 are overexpressed at high frequencies in liver tumor tissues.

In addition, we analyzed correlations between GRP75 and HSP90 expression stages and clinical-pathological stage of HCC patients. Groups 1 (+/−) and 2 (+) were considered representative of low expression and group 3 (++) and group 4 (+++) were considered representative of high expression. We found that expression of both GRP75 and HSP90 in the HCC tissues were positively correlated with the development and progression of liver cancer,since high levels of GRP75 expression were detected in 30 out of 31 tumors from T3 patients, but in only 11 out of 32 tumors from T2 patients, and high levels of HSP90 expression were detected in 28 out of 31 tumors from T3 patients, but in only 9 out 32 tumors T2 patients. These findings suggested that the increased expression of GRP75 and HSP90 in HCC tissues may play an essential role in tumorigenesis or the progression of liver tumors.

### Effects of HSP90 Inhibition on HCC Cells

**Figure 2:**
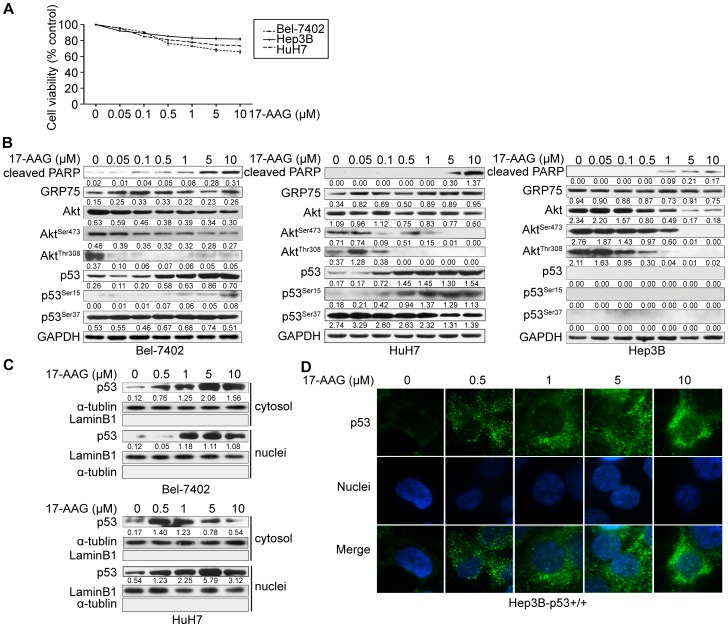
Effects of HSP90 inhibition on HCC cells. (A) HCC cell lines were treated with increasing concentrations (0.05 *µ*M to 10 *µ*M) of the HSP90 inhibitor 17-AAG for 24 hours. Cell viability was assayed using Cell Count Kit-8 (Dojindo Laboratories) and presented as relative viabilities compared to control cells exposed to vehicle (DMSO). (B) Bel-7402, HuH7 and Hep3B cells were treated with increasing concentrations of 17-AAG for 24 hours and the expression of the total Akt, phospho-Akt^Ser473^, phosphor-Akt^Thr308^, p53, phospho-p53^Ser15^, phospho-p53^Ser37^ and cleavage of PARP analyzed by western blotting. Representative results were shown (n = 3). (C) Bel-7402 (upper panel) and HuH7 (lower panel) cells, were treated with indicated concentrations of 17-AAG for 24 hours and localization of p53 was analyzed by subcellular fraction. Equal amounts of proteins from cytoplasmic and nuclear fractions were subjected to western blotting analysis using p53 antibodies.α-tublin was assayed as a loading control for the cytoplasmic fraction and Lamin B1 as a loading control for the nuclear fraction. (D) Hep3B cells infected with Lenti-p53 (Hep3B-p53^+/+^) were treated with the indicated concentrations of 17-AAG for 24 hours and immunofluorescent staining was performed to determine p53 subcellular localization. Nuclei were visualized by staining the cells with Hoechst 33258.

We first evaluated the effects of 17-AAG treatment on cell viability using a panel of HCC cell lines Bel-7402, HuH7, and Hep3B. Consistent with previous studies [30], viability of HCC cells exposed to 17-AAG (dosage from 0.05 *µ*M to 10 *µ*M) for 24 hours decreased in a concentration-dependent manner (Figure 2A).

We next examined the signaling intermediates affected by 17-AAG in Bel-7402 and HuH7 cells. Consistent with previous studies, blocking HSP90 decreased levels of total-Akt, Akt^Ser473^ phosphorylation, and Akt^Thr308^ phosphorylation, but dramatically increased p53 expression downstream from Akt in both Bel-7402 and HuH7 cells (Figure 2B). Also, we found that the mRNA (Figure S1) and protein level (Figure 2B) of GRP75 increased after inhibition of HSP90 in these cells.

Phosphorylation of p53 is known to influence p53 stability and activation [31]. To date, phosphorylation of only three p53 sites (ser15, ser37, ser392) have been detected in HCC cell lines [14]. For this reason, we investigated the phosphorylation status of p53 at ser15 and ser37 in the Bel-7402 and HuH7 cells upon exposure to 17-AAG, and found that phosphorylation of p53 at ser392 could not be detected. In contrast, we found that p53^Ser15^ phosphorylation increased in both Bel-7402 cells and HuH7 cells in a 17-AAG concentration-dependent manner (Figure 2B). Exposure to 17-AAG, however, had no detectable effect on p53^Ser37^ phosphorylation in either cell line. Increased levels of apoptosis in Bel-7402 cells and HuH7 cells in response to exposure to increasing concentrations of 17-AAG were indicated by increased levels of poly (ADP-ribose) polymerase (PARP) cleavage (Figure 2B).

It has previously been shown that 17-AAG induces tumor cell apoptosis in a p53-dependent manner [32]. We postulated that 17-AAG not only induces p53 expression, but also triggers p53 nuclear translocation. Cell lysate fractionation was performed for both Bel-7402 and HuH7 cells to determine the subcellular localization of p53. As shown in Figure 2C, exposure to 17-AAG dramatically increased the nuclear translocation of p53 in Bel-7402 and HuH7 cells. We confirmed nuclear translocation of p53 in Hep3B (p53^−/−^) by introducing a p53 expression vector into Hep3B(p53^−/−^) cells. Again, 17-AAG-mediated inhibition of HSP90 induced nuclear translocation of p53 in the cells (Figure 2D).

### Effect of Combining HSP90 and GRP75 Inhibitor on HCC Cells

**Figure 3:**
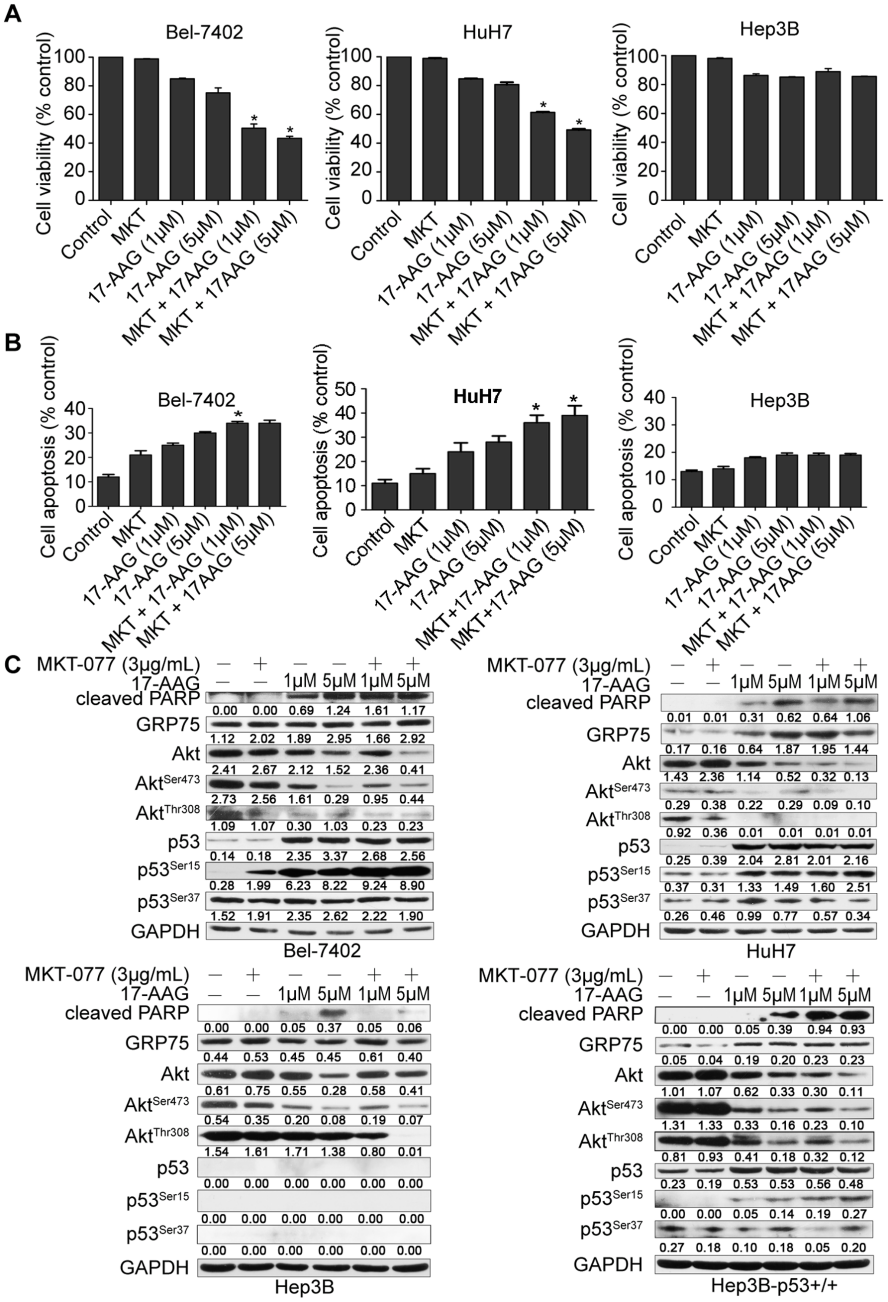
MKT-077 potentiates 17-AAG induced HCC cells apoptosis. (A) The indicated cell lines were treated with MKT-077 (MKT) or 17-AAG alone or in combination at indicated dosages for 24 hours. Cell viability was qualified using the Cell Counting Kit-8 (Dojindo Laboratories). * denotes *P* < 0.05 comparing 17-AAG (1 *µ*M or 5 *µ*M) to MKT-077 (3 *µ*g/mL) +17-AAG (1 *µ*M or 5 *µ*M). (B) Increased cell apoptosis following combined treatment. Bel-7402, HuH7 and Hep3B cells treated as described above were subjected to Annexin-V and PI staining and cell apoptosis was quantified by FACS. * denotes *P* < 0.05 comparing 17-AAG (1 *µ*M or 5 *µ*M) to MKT-077 (3 *µ*g/mL) +17-AAG (1 *µ*M or 5 *µ*M). (C) Levels of cleaved PARP, GRP75, Akt, phospho-Akt^Ser473^, phosphor-Akt^Thr308^, p53, phospho-p53^Ser15^, and phospho-p53^Ser37^ were detected by Western Blotting analysis. GAPDH served as a loading control.

MKT-077, an inhibitor of GRP75, has been shown to disrupt GRP75-p53 complexes, thereby facilitating p53 nuclear translocation and rescuing p53 function [27]. For this reason, we hypothesized that combinations of MKT-077 may strengthen the effects of 17-AAG on HCC cells. To test this hypothesis, we examined cell viability and apoptosis for cells exposed to MKT-077 (3 *µ*g/mL) or 17-AAG (1 *µ*M and 5 *µ*M) alone or in combination. After 24 h treatment, cell viability was measured by MTT assay and cell apoptosis was analyzed by flow cytometry. As shown in Figure 3A and B, Bel-7402 or HuH7 cells treated with both agents showed significant decreases in cell viability and increases cell apoptosis compared with cells treated with either compound alone (Figure S2). In contrast, exposure to MKT-077 did not increase the effects of 17-AAG on cells viability or apoptosis in Hep3B cells (Figure 3B), which lack endogenous p53.

We next measured levels of Akt and, p53, and their phosphorylation status in Bel-7402, HuH7, Hep3B and Hep3B (p53^+/+^) cells in response to single or combined treatment with 17-AAG and MKT-077. In the presence of MKT-077, 17-AAG decreased levels of Akt protein as well as levels of phosphorylated Akt^Ser473^ and Akt^Thr308^ in these cells relative to cells treated with 17-AAG alone. In contrast, in the presence of MKT-077, the expression of the Akt downstream effecter p53 in each of the cell lines did not increase, but p53^Ser15^ phosphorylation was further induced by MKT-077 addition in Bel-7402, HuH7 and Hep3B (p53^+/+^) cells. However, p53 phosphorylation at Ser37 was not affected by any agent combination in either cell line (Figure 3C). In addition, PARP cleavage, which is an indicator of apoptosis, increased in cells subjected to treatment with both agents in Bel-7402, Huh7 and Hep3B (p53^+/+^) cells (Figure 3C). These data suggested that MKT-077 may improve the anti-tumor efficacy of 17-AAG by blocking Akt activation, and by stabilizing and activating p53.

### MKT-077 Promotes the Nuclear Translocation of p53 Induced by17-AAG in HCC Cells

**Figure 4:**
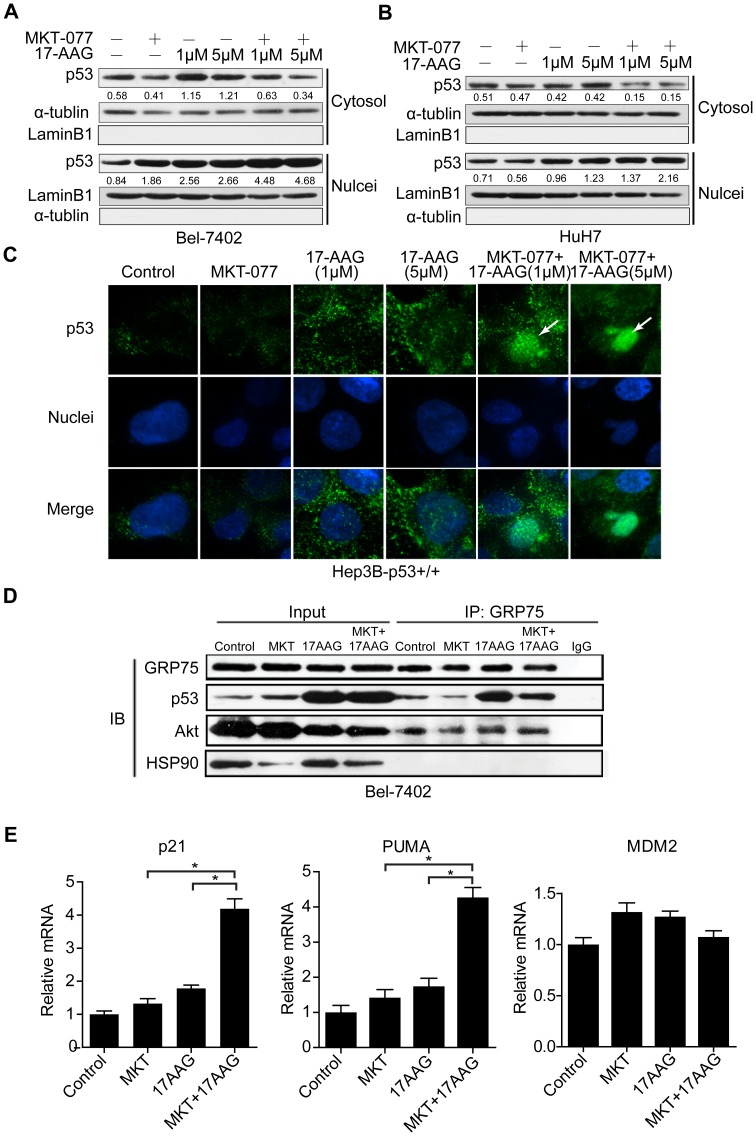
MKT-077 potentiates 17-AAG-induced translocation of p53 to the nuclei in HCC cells. (A, B) Bel-7402 and HuH7 cells were treated with MKT-077, 17-AAG or a combination of both agents at the indicated concentrations for 24 hours. Cytoplasmic and nuclear fractions of Bel-7402 and HuH7 cells were separated, and subjected to Western blotting analysis of p53 expression. α-tublin and Lamin B1 served as loading controls for cytoplasmic and nuclear fractions respectively. (C) Hep3B cells infected with Lenti-p53 (Hep3B-p53^+/+^) were treated with MKT-077, 17-AAG or MKT-077+17-AAG at indicated dosages for 24 hours, and immunofluorescent staining was performed to determine p53 subcellular localization. (D) Bel-7402 cells treated with MKT-077 or 17-AAG alone, or MKT-077+17-AAG for 24 hours were harvested and subjected to co-immunoprecipitation using GRP75-specific antibodies. Levels of p53, Akt and HSP90 were detected by Western blotting analysis. (E) MKT-077 and 17-AAG synergistically enhanced mRNA levels of the p53 target genes *p21* and *PUMA*, and reduced *MDM2* mRNA levels. The mRNA levels of these genes were quantified using real-time RT-PCR and normalized to levels of GAPDH mRNA. * denotes *P* < 0.05 comparing 17-AAG to MKT-077+17-AAG.

Next, we examined how MKT-077 modulates 17-AAG-activated p53 to explain the synergism observed in the promotion of 17-AAG-induced apoptosis in HCC cells. We first determined the subcellular localization of endogenous p53 in Bel-7402 and HuH7 cells. We analyzed levels of p53 in the cytoplasm and nuclear fractions of Bel-7402 (Figure 4A) and HuH7 cells (Figure 4B). These measurements showed that combinations of 17-AAG and MKT-077 promoted translocation of endogenous p53 from the cytoplasm to the nuclei to a greater extent than 17-AAG or MKT-077 alone. To confirm these results, we transfected Hep3B cells with GFP-tagged p53 and examined its subcellular localization. As in the case of Bel-7402 cells, GFP-tagged p53 showed intense nuclear staining in cells exposed simultaneously to both inhibitors (Figure 4C).

Previous studies have shown that the formation of the GRP75-p53 complex can inhibit nuclear translocation of p53 and thereby hinder p53-dependent apoptosis in tumor cells [33]. We confirmed the interaction between GRP75 and p53 in untreated Bel-7402 cells. Exposure to 17-AAG greatly increased binding between GRP75 and p53, but this interaction was significantly impaired by co-treatment with MKT-077 (Figure 4D). We also found that GRP75 coimmunoprecipitates with Akt, but this interaction was not affected by any of the above treatments. In contrast, HSP90 bound to neither GRP75 nor p53 in HCC cells (Figure 4D). We next examined gene expression of three classical p53 targets: i) p21, ii) p53 upregulated modulator of apoptosis (PUMA) and iii) the p53 inhibitory regulator MDM2 (murine double minute-2). As shown in Figure 4E, expression of p21 and PUMA was synergistically increased in the presence of both MKT-077 and 17-AAG, but the levels of MDM2 were reduced following exposure to the combination of inhibitors. Thus, MKT-077 combined with 17-AAG not only blocks Akt activation and stabilizes p53, but also inhibits sequestration of p53 by GRP75, thus promoting p53 accumulation in the nuclei. These data imply that MKT-077 enhances activation of transcription by p53 and increases the rate of 17-AAG-induced apoptosis in HCC cells.

### Combination of HSP90 and GRP75 Inhibitors for HCC Therapy *in vivo*

**Figure 5:**
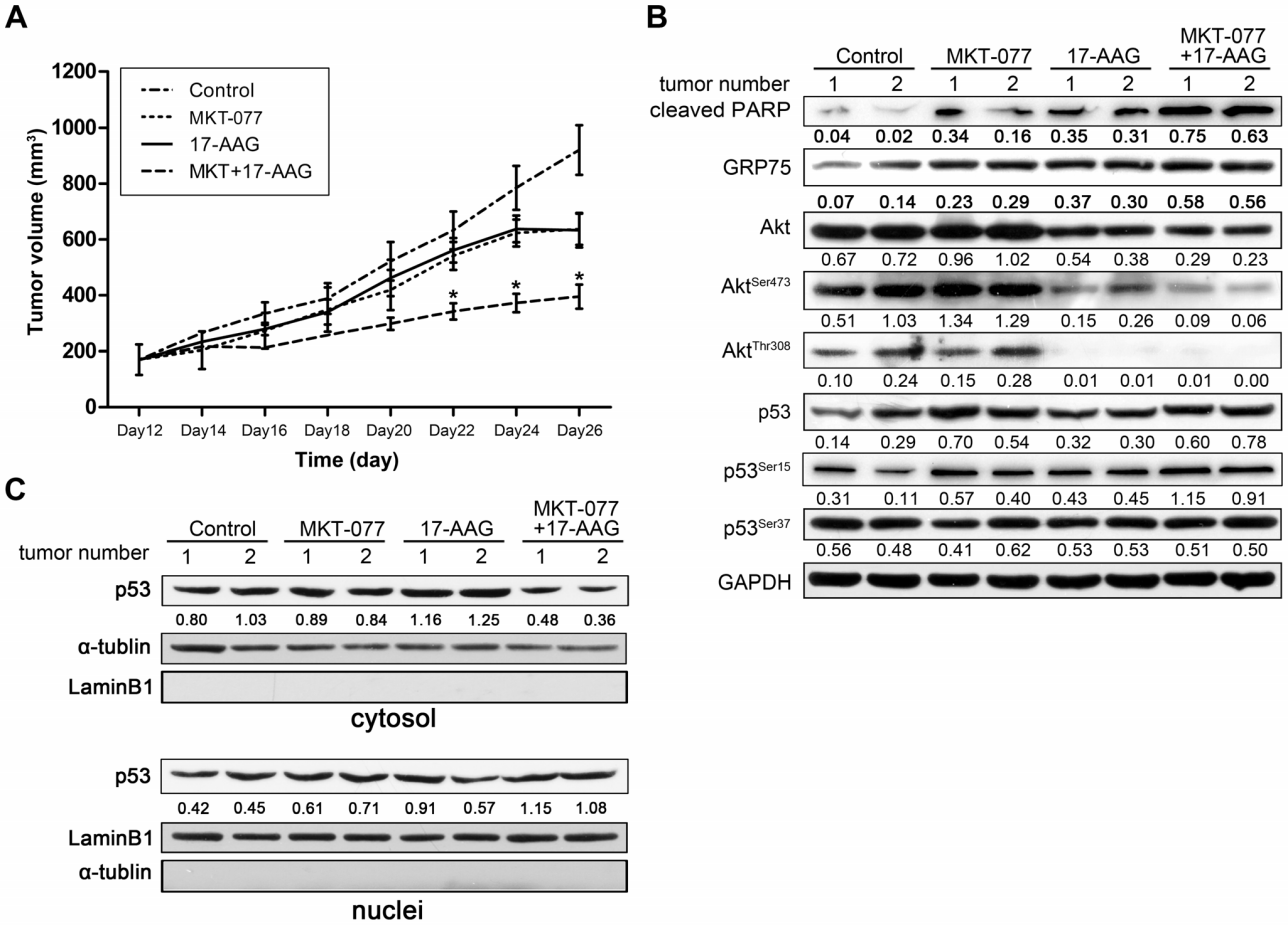
Dual inhibition of HSP90 and GRP75 inhibit tumor growth *in vivo*. (A) Bel-7402 cells (5×10^6 ^per flank) were injected into the right flank of nude mice. Treatment started after 12 days when tumors had reached 200–300 mm^3^ in volume. Mice were randomly divided into four groups (n = 5 each): control mice exposed to vehicle (physiological saline), MKT-077 alone, 17-AAG alone, or MKT-077+17-AAG. The graph shows tumor volume measured every 2 days. Data are presented as the mean ± SEM. * *P* < 0.05. (B) The indicated proteins in tumor tissues were analyzed by western blotting. Representative results are shown. (C) Cytoplasmic and nuclear fractions of tumor tissues were separated, and subjected to Western blotting analysis of p53 expression. α-tublin and Lamin B1 served as loading controls for cytoplasmic and nuclear fractions respectively.

To further determine the anti-hepotocarcinogenic efficacy of the HSP90 and GRP75 inhibitors *in vivo*, we subcutaneously implanted human hepatocellular Bel-7402 cells into nude mice. As shown in Figure 5A, the growth of subcutaneous tumors in mice treated with both MKT-077 and 17-AAG was markedly lower than that of control mice or mice treated with either inhibitor alone (Figure 5A). Tumors in mice given the combination of inhibitors remained stable after day 16, but tumor grow accelerated in mice treated with either agent alone. Western blotting analysis was used to monitor the levels of the indicated proteins (Figure 5B). Consistent with results observed *in vitro*, activation of Akt, measured by levels of Akt^Ser473^ and Akt^Thr308^ phosphorylation, was blocked by 17-AGG alone and by combined exposure to 17-AGG and MKT-077 *in vivo*. In contrast, exposure to 17-AGG and MKT-077 increased levels of p53^Ser15^ phosphorylation. Increased tumor cell apoptosis, measured by increased levels of cleaved PARP, was also observed in response to combined inhibition of HSP90 and GRP75 (Figure 5B). Although increased GRP75 levels induced by 17-AAG might favor HCC cell growth, combination treatment was found to enhance p53 nuclear translocation (Figure 5C). Thus, combined inhibition of HSP90 and GRP75 was found to block Akt activation, facilitate p53 accumulation in the nuclei and activate growth-inhibitory target genes, ultimately reduce HCC tumor growth *in vivo*.

## Discussion

Liver cancer, one of the most common and lethal malignancies, is a significant threat to human health and is greatly in need of novel therapeutic agents and new therapeutic strategies. HSP90 has been shown to be a promising anti-cancer target in cancer therapy [34]. HSP90 inhibitors act by blocking the formation of complexes with multiple client proteins that contribute to tumorigenesis and cell growth, thereby blocking several distinct signaling pathways related to cell survival [35], [36]. The HSP90 inhibitor 17-AAG has been proven to have potent anti-tumor effects, but it can also promote the expression of compensatory HSPs that favor cell proliferation and survival [35]. As a consequence, these partially reduce 17-AAG’s anticancer effects. For example, upon 17-AAG treatment, the expression of HSP70 was increased and elevated expression of HSP70 has been implicated in contributing to cancer cell survival via multiple anti-apoptotic pathways [37]. A recent study showed that dual silencing HSC70 and HSP70 dramatically increase the rate of HSP90-inhibitor-induced tumor-specific apoptosis [37]. In the present study, we confirmed that the expression level of GRP75, another member of HSP70 family proteins, was also increased following HSP90 inhibition with 17-AAG. It has been shown that overexpression of GRP75 can protect cells under stressed conditions [14]. Thus, elevated levels of GRP75 expression induced by 17-AAG in turn attenuated the growth-inhibitory effect of 17-AAG on cancer cells. In this study, we demonstrated that dual targeting GRP75 and HSP90 can also confer potent anti-tumor effect against cancer, which provide one more therapeutic strategy for liver cancer therapy in addition to dual inhibition of HSP70 and HSP90.

The rationale for combining GRP75 inhibitors and HSP90 inhibitors is based on the following observations. First, inhibition of HSP90-induced cell death partly depends on p53 signaling pathway [11]. Second, overexpression of GRP75 can lead to permanent sequestration of p53 in the cytoplasm, resulting in inhibition of its transcriptional activation. Inhibition of GRP75 disturbs the GRP75-p53 complex, causing p53 to accumulate in the nuclei, where it induces the expression of apoptosis-related genes [33], [38]. Third, the inhibition of GRP75 can block the activation of Akt which thereby leads to p53 activation [39]. Fourth, elevated expression of HSP90 and GRP75 has frequently been observed in HCC tissues and these increased expression levels are closely correlated with advanced pathologic stages of cancer (Figure 1A). Based on the fact that induction of apoptosis is one of the most important strategies in anti-tumor therapy [40] and that both 17-AAG and MKT-077 exert their anti-cancer effects in a p53-dependent manner, we proposed that combinations of HSP90 inhibitors and the GRP75 inhibitors could be significantly and synergistically effective in HCC therapy.

Akt, which mediates signaling pathways downstream of activated tyrosine kinase and PI3K, regulates a wide range of cellular functions, including cell proliferation and survival [41]. The activation of Akt depends on its phosphorylation states. Akt can be phosphorylated on two sites: Thr308 and Ser473, both of which do not depend on one another [42]. Akt is an upstream role of p53 and phosphorylated Akt can interrupt stability and activity of p53 [42]. Our study confirmed that 17-AAG reduces total Akt protein levels and levels of Akt phosphorylation at Ser473 and Thr308 (Figure 2B) in liver cancer cells. We also showed that combinations of MKT-077 and 17-AAG, further reduced levels of phospho-Akt Ser473 and Thr308 (Figure 3C).

p53-mediated apoptosis has been shown to be one mechanism of tumor suppression, and hyperactivation of p53 is lethal to cancer cells [43]. Previous studies have revealed that HSP90 inhibitors can preserve the stability and activation of p53 by promoting the phosphorylation of p53 in mantle cell lymphoma [44]. In this study, we found that inhibition of HSP90 upregulated p53 and enhanced p53 phosphorylation at ser15 but had no effect on ser37 phosphorylation. These data may suggest that phosphorylation of p53 at ser15 is much more important than phosphorylation at ser37 for determining the sensitivity of HCC cells to 17-AAG.

Although, mutation in p53 is frequently found in liver tumors, the major of p53 mutation observed in HCC is at the third position of codon 249 resulting in a G:C to T: A transversion [45]–[48]. Lu et. have evidenced that the inhibition of GRP75 can still strongly reactivate the apoptotic function of mutant p53 in HCC cells [49]. Thus, such therapeutic strategy that dual targeting of GRP75 and HSP90 will be excepted to be effective in part HCC which harboring mutant p53.

Finally, our study shows that HCC cell proliferation is inhibited to a significant extent by combined treatment with 17-AAG+MKT-077 *in vivo*. Previous clinical studies have shown that animals given high doses of MTK-077 eventually experienced irreversible renal toxicity. For this reason, we modified our treatment schedule, using doses of 3 mg/kg every other day. This dosage is considered low and has been shown to have no toxicity in murine models [50], but effective in releasing p53 from GRP75-p53 complexes.

Taken together, our data evidenced that the HSP90 inhibitor 17-AAG reduces the level and activity of Akt, increases expression of p53 protein and stabilizes p53 by enhancing p53 phosphorylation. Although the HSP90 inhibitor 17-AAG could induce p53 expression, most of the induced p53 is sequestrated in the cytoplasm by forming GRP75-p53 complex. However, the addition of MKT-077 disrupts interactions between GRP75 and p53 and releases p53 for translocation to the nuclei. This may explain how the addition of MKT-077 to 17-AAG stimulates p53 nuclear translocation and the expression of p53-dependent apoptosis genes, including p21 and PUMA. Our data show that although the level of endogenous p53 is not higher in HCC cells subjected to combination treatment than treated with 17-AAG alone, the increased level of apoptosis might be attributable to increased nuclear translocation of p53. Our study provides preclinical evidence for combination therapy with GRP75 inhibitor and HSP90 inhibitor for enhanced killing of HCC cells.

## Supporting Information

Figure S1 **17-AAG induced mRNA levels of GRP75 in a dose-dependent manner.** Bel-7402 cells were treated with increasing concentrations of 17-AAG (0.05 µM-10 µM) for 24 hours. Cells were harvested; total RNA was extracted and subjected to subsequent quantitative RT-PCR analysis of *GRP75* mRNA. Data were normalized to levels of GAPDH mRNA. Results are representative of three independent experiments.
(TIF)

Figure S2 **Increased cell apoptosis following 17-AAG+MKT-077 treatment.** Bel-7402 and Hep3B cells were treated with MKT-077 (MKT) or 17-AAG alone or in combination at indicated dosages for 24 hours, and then subjected to Annexin-V and PI staining. Cell apoptosis was quantified by FACS. The percentage of total apoptotic cells was shown at the upper-right corner of each panel.
(TIF)
