# Supplementary figures and images for: Targeting GRP75 Improves HSP90 Inhibitor Efficacy by Enhancing p53-Mediated Apoptosis in Hepatocellular Carcinoma
Source: PLoS One. 2014 Jan 17;9(1):e85766. doi: 10.1371/journal.pone.0085766 (PMC3894982; doi:10.1371/journal.pone.0085766)

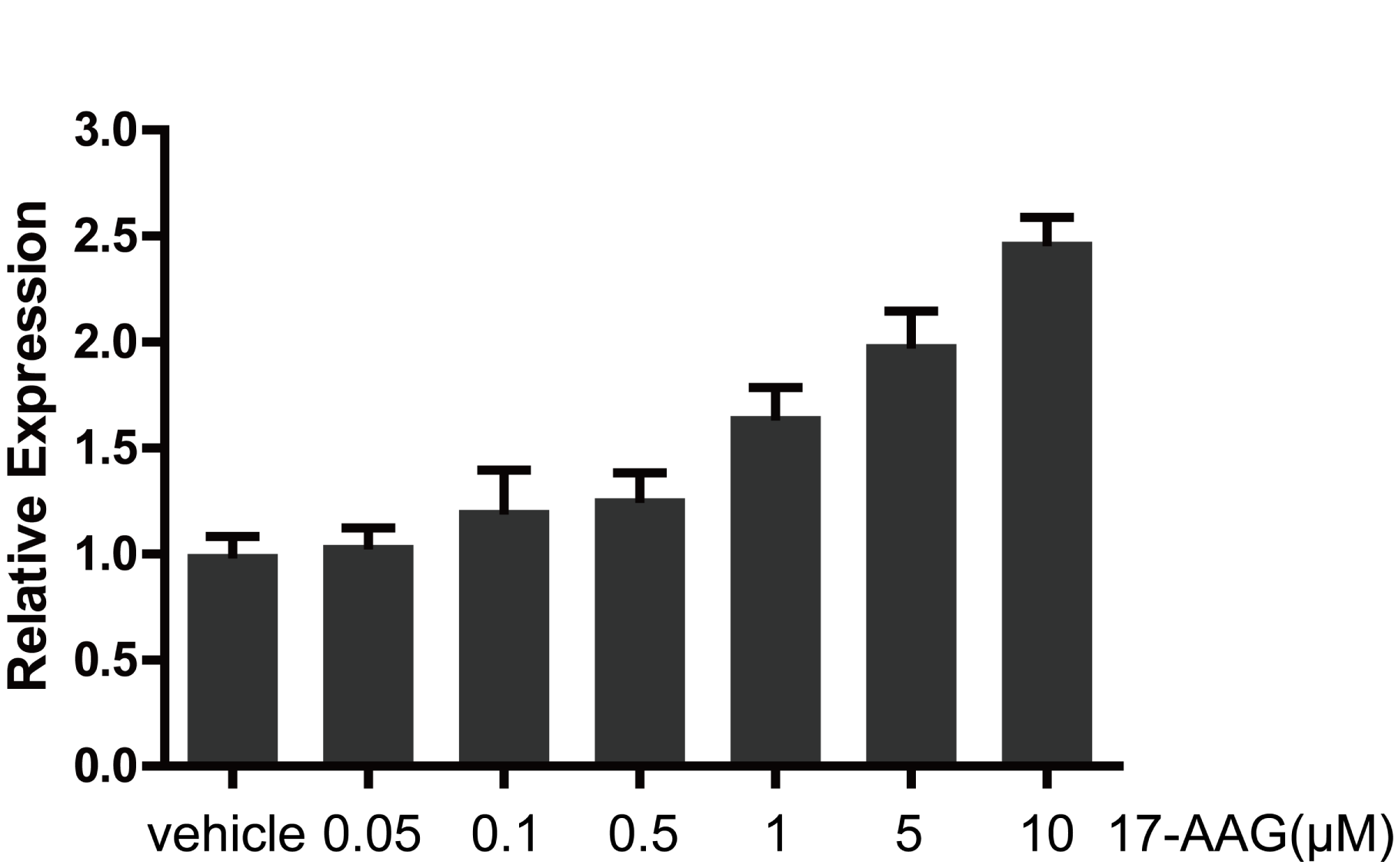

Supplement: Figure S1 — 17-AAG induced mRNA levels of GRP75 in a dose-dependent manner. Bel-7402 cells were treated with increasing concentrations of 17-AAG (0.05 µM-10 µM) for 24 hours. Cells were harvested; total RNA was extracted and subjected to subsequent quantitative RT-PCR analysis of GRP75 mRNA. Data were normalized to levels of GAPDH mRNA. Results are representative of three independent experiments. (TIF) [file pone.0085766.s001.tif]

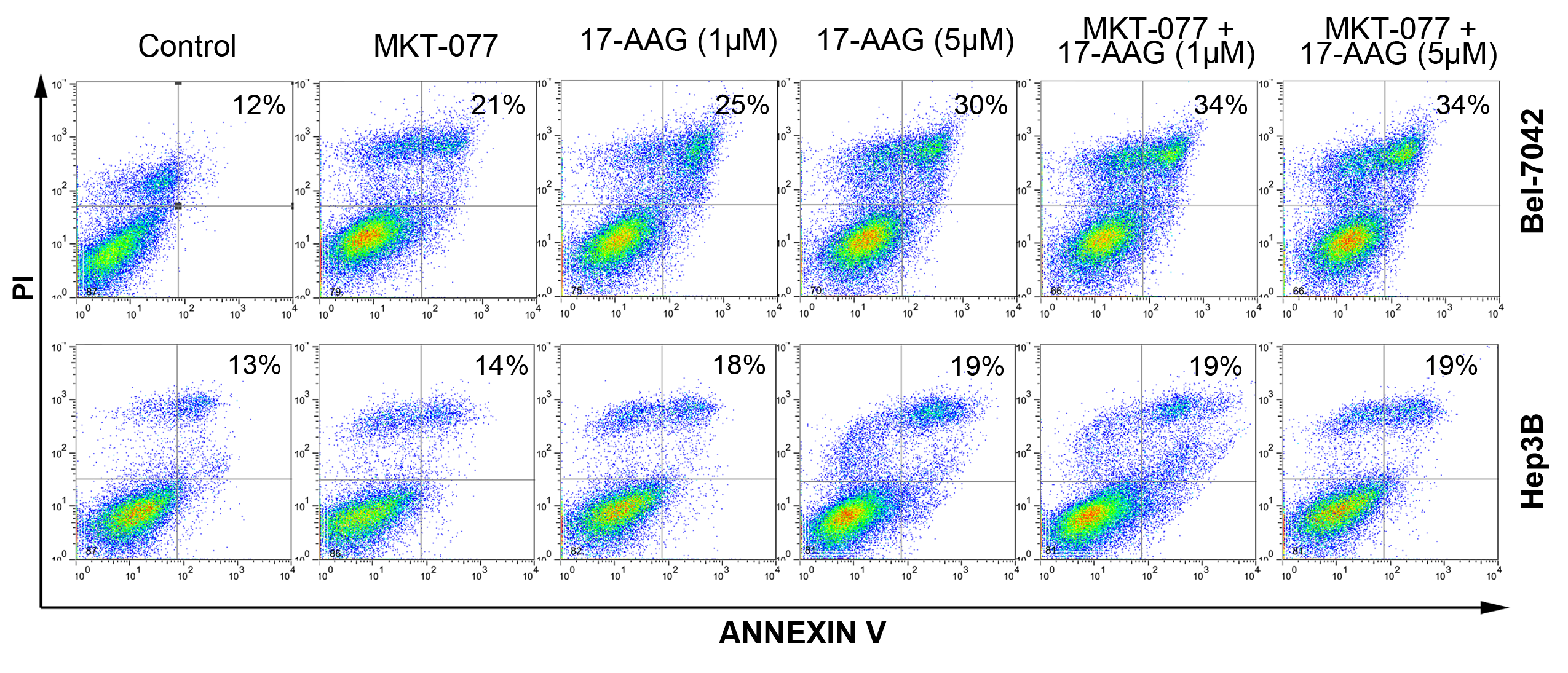

Supplement: Figure S2 — Increased cell apoptosis following 17-AAG+MKT-077 treatment. Bel-7402 and Hep3B cells were treated with MKT-077 (MKT) or 17-AAG alone or in combination at indicated dosages for 24 hours, and then subjected to Annexin-V and PI staining. Cell apoptosis was quantified by FACS. The percentage of total apoptotic cells was shown at the upper-right corner of each panel. (TIF) [file pone.0085766.s002.tif]
